# Trehalose 6‐phosphate – a central regulator at the crossroads of sugar signalling, metabolism, and development

**DOI:** 10.1111/nph.70533

**Published:** 2025-09-08

**Authors:** Franziska Fichtner

**Affiliations:** ^1^ Institute of Plant Biochemistry and Cluster of Excellences on Plant Science (CEPLAS) Heinrich Heine University Düsseldorf, Faculty of Mathematics and Natural Sciences Düsseldorf 40225 Germany

**Keywords:** plant metabolic regulation, single cell sequencing, SnRK1, spatial regulation of metabolism, sucrose, sugar signalling, trehalose 6‐phosphate

## Abstract

In mammals, blood sugar levels are tightly controlled by two hormones: insulin and glucagon. In flowering plants, a comparable regulatory mechanism exists, mediated by the sugar‐signalling molecule trehalose 6‐phosphate (Tre6P). Similar to insulin, Tre6P functions as a signal and negative feedback regulator of sucrose, the main transport sugar in vascular plants. In the model plant *Arabidopsis thaliana* and likely all other angiosperms, Tre6P is predominantly synthesized in the vasculature, an ideal position to integrate systemic sugar status with whole‐plant developmental decision‐making. Genes encoding components of Tre6P dephosphorylation and signalling show broader expression patterns suggesting movement and signalling of Tre6P outside the vasculature to coordinate plant metabolism and development.

**Table:** 

	Contents	
	Summary	2243
I.	Introduction	2243
II.	Discovery and function of trehalose metabolism in plants	2243
III.	Tre6P is a sucrose‐specific signal	2244
IV.	Metabolic regulation by Tre6P	2244
V.	Tre6P as a key signal in regulating development	2244
VI.	A Tre6P‐SnRK1 nexus?	2245
VII.	The mystery of spatial regulation of Tre6P metabolism and signalling	2245
VIII.	Intracellular localization of Tre6P signalling	2248
IX.	Conclusion	2248
	Acknowledgements	2248
	References	2248

## I. Introduction

As sessile organisms, plants continuously monitor and manage internal resources to optimize growth. They fix CO_2_ from the atmosphere to produce sugars, which serve as carbon and energy sources, and as signalling molecules. Among these, sucrose is one of the main products of photosynthesis and the main sugar transported from source to sink organs in vascular plants. Trehalose 6‐phosphate (Tre6P) functions as a sucrose‐specific signal and central regulator of sucrose levels, integrating metabolic cues with developmental processes.

## II. Discovery and function of trehalose metabolism in plants

**Fig. 1:**
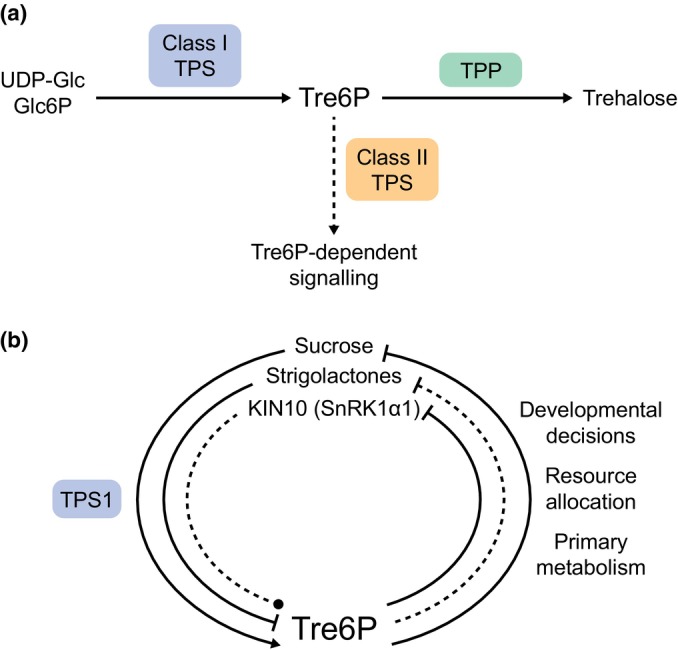
The trehalose 6‐phosphate (Tre6P) signalling nexus. (a) Tre6P is synthesized by catalytically active class I Tre6P synthases (TPS, blue) and dephosphorylated by Tre6P phosphatases (TPP, green). Catalytically inactive class II TPSs (orange) are likely involved in downstream Tre6P signalling. (b) Tre6P levels are influenced by sucrose levels and strigolactones and might involve KIN10. This involves regulation of the TPS1 enzyme (blue), the predominant TPS. Tre6P can inhibit sucrose and KIN10 by different means. Via activation of organic acid synthesis, Tre6P might also inhibit strigolactone signalling. Dashed lines represent hypothetical interactions while solid lines are based on published studies.

Tre6P is the intermediate of trehalose biosynthesis, which is conserved across the plant kingdom (Lunn *et al*., 2014). Trehalose (α‐d‐glucopyranosyl‐[1,1]‐α‐d‐glucopyranoside) is found in all domains of life, except for vertebrates, and is used as the main transport sugar in insects. Historically, trehalose detection in plants was dismissed as microbial contamination, except in ‘resurrection plants’ that accumulate trehalose under drought (Lunn *et al*., 2014). This assumption changed with the characterization of transgenic plants expressing trehalose pathway genes from *Escherichia coli*, which showed striking phenotypes suggesting an active role for trehalose metabolism in plants. In eukaryotes, trehalose is synthesized exclusively via Tre6P, which is synthesized by Tre6P synthases (TPS), and dephosphorylated to trehalose by Tre6P phosphatases (TPP; Fig. 1a; Fichtner & Lunn, 2021).

## III. Tre6P is a sucrose‐specific signal

Tre6P is present at very low concentrations (in the low μM range), and its quantification was initially challenging until the development of a highly specific and sensitive LC‐MS/MS‐based assay (Lunn *et al*., 2006). Its hormone‐like concentration supports the idea that Tre6P functions primarily as a signalling molecule, rather than serving a metabolic function.

Feeding experiments in arabidopsis showed that Tre6P correlates most strongly with sucrose rather than with its own precursors (Lunn *et al*., 2006; Yadav *et al*., 2014). This correlation is thereby conserved across angiosperms (Annunziata *et al*., 2025). Furthermore, transgenic lines overexpressing bacterial TPS or TPP enzymes altered Tre6P:sucrose ratios but did not break the tight correlation between the two (Yadav *et al*., 2014). These data gave rise to the conceptual framework of a sucrose–Tre6P nexus, proposing that Tre6P acts as a signal of sucrose status and a homeostatic feedback regulator (Fig. 1b; Figueroa & Lunn, 2016; Fichtner & Lunn, 2021).

## IV. Metabolic regulation by Tre6P

To avoid pleiotropic effects of constitutive TPS and TPP overexpression, inducible systems were used to investigate the direct downstream consequences of Tre6P accumulation. Increased Tre6P levels in arabidopsis rosettes reduced sucrose levels, while increasing tricarboxylic acid (TCA) cycle intermediates and amino acids (Figueroa *et al*., 2016). A similar metabolic shift was detected when Tre6P levels increased in sink tissues like axillary buds of garden pea (*Pisum sativum*) after decapitation (Fichtner *et al*., 2017). These data imply that Tre6P modifies metabolism to modulate sucrose levels to prime tissues for growth (Fig. 1b). Tre6P also suppresses starch degradation at night or at the end of a long day, likely by inhibiting an early stage of starch breakdown (Martins *et al*., 2013; Ishihara *et al*., 2022).

Besides this function in modulating sucrose partitioning, Tre6P influences sucrose allocation by modulating the expression of *SUGARS WILL EVENTUALLY BE EXPORTED TRANSPORTERS* (*SWEETs*), which mediate sucrose loading in source tissues and unloading in sinks (Chen *et al*., 2012; Oszvald *et al*., 2018; Fichtner *et al*., 2021b; Avidan *et al*., 2024). For example, overexpressing a *TPP* in maize ears lowered Tre6P levels but elevated sucrose levels, ultimately increasing yield under well‐watered and drought conditions (Nuccio *et al*., 2015). Elevated Tre6P levels also enhance starch and fatty acid biosynthesis in developing seeds of arabidopsis, pea, and wheat, likely impacting seed filling and yield (Martínez‐Barajas *et al*., 2011; Griffiths *et al*., 2016, 2025; Zhai *et al*., 2018; Meitzel *et al*., 2021).

## V. Tre6P as a key signal in regulating development

Tre6P plays a central role in plant development. Schluepmann *et al*. (2003) first demonstrated that altering Tre6P levels causes striking developmental phenotypes like early flowering and altered leaf development. Subsequent studies confirmed both local (within meristems) and systemic roles of Tre6P in regulating flowering and shoot branching in arabidopsis, pea, and maize (Fichtner & Lunn, 2021). This involves activation of *FLOWERING LOCUS T* (*FT*) and *SWEETs* by Tre6P (Wahl *et al*., 2013; Oszvald *et al*., 2018; Fichtner *et al*., 2021a; Zacharaki *et al*., 2022).

Besides activation of FT and sugar transporters, Tre6P signalling is also linked to hormones like abscisic acid (reviewed in Fichtner & Lunn, 2021), or strigolactones (see following section). Strigolactones control a large variety of different developmental processes, including inhibition of shoot branching (Dun *et al*., 2023). Tre6P acts downstream of strigolactones to modulate bud outgrowth (Fichtner *et al*., 2024). The F‐box protein MORE AXILLARY GROWTH2 (MAX2), a key component of strigolactone signalling, is inhibited by citrate, a TCA cycle intermediate (Tal *et al*., 2022). Since Tre6P enhances carbon flux into the TCA cycle and its synthesis is inhibited by strigolactones (Fichtner *et al*., 2024), this suggests a negative feedback loop between Tre6P, metabolism, and strigolactone signalling (Fig. 1b; Barbier *et al*., 2023). In arabidopsis, Tre6P regulation by strigolactones occurs independently of the transcription factor BRANCHED1 (BRC1; Fichtner *et al*., 2021a), unlike in maize, where TEOSINTE BRANCHED1 (the BRC1 homologue in monocots) probably directly regulates Tre6P degradation (Dong *et al*., 2019; Klein *et al*., 2022).

In arabidopsis, Tre6P has also been implicated in the modulation of root growth, especially lateral root development (Fichtner *et al*., 2020; Muralidhara *et al*., 2021; Lin *et al*., 2022; Morales‐Herrera *et al*., 2023). Under phosphate deficiency, Xia *et al*. (2025) found companion cell‐specific regulation of genes involved in Tre6P metabolism. Similarly, Tre6P may integrate with nitrate signalling at the shoot apex (Olas *et al*., 2019), linking Tre6P signalling to nutrient‐dependent growth regulation.

## VI. A Tre6P‐SnRK1 nexus?

The protein kinases Sucrose non‐Fermenting‐1‐Related Kinase 1 (SnRK1) and TARGET OF RAPAMYCIN (TOR) are major antagonistic regulators of growth – SnRK1 promotes energy conservation, while TOR drives growth activation (Margalha *et al*., 2019). As growth requires substantial energy investment, sugar signalling pathways via Tre6P, SnRK1, and TOR converge to efficiently align metabolic status with developmental cues. In the following, the focus is on the link between Tre6P and SnRK1. For detailed discussions on the link between Tre6P and TOR, the reader is referred to more extensive reviews (Caldana *et al*., 2023; Gobel & Fichtner, 2023; Morales‐Herrera *et al*., 2024).

The SnRK1 complex comprises a catalytic α subunit (KIN10‐12) and regulatory (β1‐3 and βγ/SNF4) subunits (Broeckx *et al*., 2016). Tre6P inhibits KIN10 (SnRK1α1) activity *in vitro* (Fig. 1b), likely by binding to its T‐loop, preventing phosphorylation by SnRK1‐ACTIVATING KINASEs (SnAKs; Zhang *et al*., 2009; Zhai *et al*., 2018; Blanford *et al*., 2024). Since KIN10 can autophosphorylate (Ramon *et al*., 2019) and its inhibition by Tre6P may involve a tissue‐specific intermediary absent in mature leaves (Zhang *et al*., 2009; Van Leene *et al*., 2022), the precise mechanism remains elusive.

Genetic data support a link between Tre6P and SnRK1: mutations in *KIN10* and *SNF4* rescue embryo‐lethal *tps1* null mutants (Zacharaki *et al*., 2022), and Tre6P and SnRK1 often show antagonistic effects. For example, low light or darkness reduced Tre6P levels, increased SnRK1 marker gene expression, and promoted lateral root initiation (Muralidhara *et al*., 2021). By contrast, in continuous light elevated Tre6P levels or mutations in *kin10*/*kin11* enhanced lateral root density, while KIN10/KIN11 overexpression suppressed it, and Tre6P could not promote branching in SnRK1 gain‐ or loss‐of‐function lines (Morales‐Herrera *et al*., 2023). A similar antagonism was demonstrated to modulate hypocotyl elongation (Hwang *et al*., 2019), suggesting Tre6P acts upstream of SnRK1.

Because *kin10,11* double mutants are embryo‐lethal, *kin10* ^−/−^ *kin11* ^−/+^ lines were used to study metabolic phenotypes. These plants had lower Tre6P and sucrose levels, while KIN10 overexpression increased both (Peixoto *et al*., 2021), suggesting feedback regulation of Tre6P synthesis by KIN10 (Fig. 1b). Transcriptomic profiling after a transient fourfold increase in Tre6P levels revealed widespread transcriptional reprogramming (> 13 000 genes), including repression of *c*. 500 SnRK1 starvation‐responsive genes, suggesting action of Tre6P partially by inhibition of SnRK1 (Avidan *et al*., 2024). Yet, Tre6P‐SnRK1 interactions *in planta* remain enigmatic. Tre6P does not always correlate with *in vivo* SnRK1 activity (Avidan *et al*., 2023), and Tre6P can even activate KIN10 *in vitro* in the absence of SnAKs (Blanford *et al*., 2024). All in all, a Tre6P‐SnRK1 nexus is likely to operate in plants; however, the specific tissues and developmental stages in which it functions remain to be elucidated.

## VII. The mystery of spatial regulation of Tre6P metabolism and signalling

In arabidopsis, the *TPS* gene family comprises 11 members divided into two classes: class I TPSs (TPS1–4) are catalytically active, and class II TPSs (TPS5‐11) with mutations in their catalytic triad are catalytically inactive in synthesizing Tre6P (Fig. 1a; Fichtner & Lunn, 2021). TPS1 is the predominant active TPS across plant species. Complementation of embryo lethal *tps1* null mutants with *E. coli* TPS restored viability, confirming Tre6P as a key factor in embryo development (van Dijken *et al*., 2004; Fichtner *et al*., 2020). AtTPS1 is primarily localized in vascular tissues, meristems, guard cells, and pollen (Fichtner *et al*., 2020), placing Tre6P synthesis at key source–sink and developmental interfaces. This includes the vasculature of developing flowers, particularly the unloading zones (Fichtner *et al*., 2020). In line with a role for Tre6P in flower development, Groh *et al*. (2025) identified a *TPP* in walnut to repress male flower development likely by lowering Tre6P levels.

**Fig. 2:**
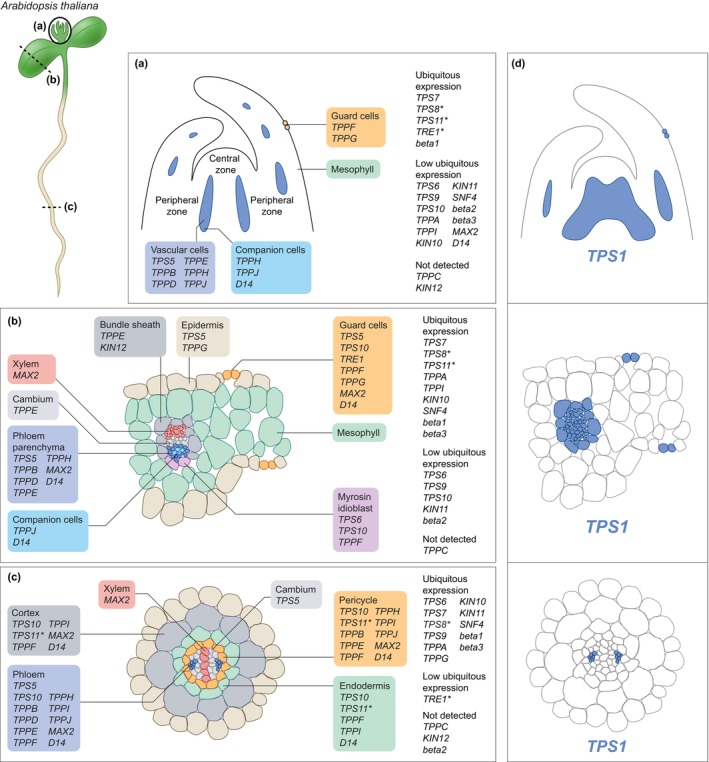
Spatial expression patterns of genes involved in trehalose 6‐phosphate (Tre6P) biosynthesis and signalling in *Arabidopsis thaliana*. (a) Single‐cell RNA‐seq studies of arabidopsis shoot apical meristems (Zhang *et al*., 2021), (b) leaves (Kim *et al*., 2021; Tenorio Berrío *et al*., 2025), and (c) roots (Fig. 2c; Denyer *et al*., 2019), and (d) the Tre6P synthase 1 (*TPS1*) expression pattern (blue colour) based on transcriptomic studies and localization of TPS1 fusion protein constructs in the *tps1‐1* knockout driven by the natural *TPS1* promoter and terminator as described in Fichtner *et al*. (2020). Genes involved in Tre6P signalling include components of strigolactone signalling (D14, DWARF14; MAX2, MORE AXILLARY GROWTH2) and the SnRK1 (Sucrose non‐Fermenting‐1‐Related Kinase 1) complex (KIN10‐12, beta1‐3, SNF4). TPP, Tre6P phosphatase; TRE1, TREHALASE1.

Meta‐analysis of single‐cell RNAseq studies of arabidopsis shoot apical meristems (Fig. 2a; Zhang *et al*., 2021), leaves (Fig. 2b; Kim *et al*., 2021; Tenorio Berrío *et al*., 2025), and roots (Fig. 2c; Denyer *et al*., 2019) confirmed expression of *AtTPS1* in vascular and meristematic tissues, and guard cells (Fig. 2d). *TPS1* mRNA was also identified in vascular tissues by single‐cell RNA sequencing of wheat (*Triticum aestivum*) roots, and *Prunus mume* petals (Zhang *et al*., 2023; Guo *et al*., 2024), and CsTPS1 was found in cucumber (*Cucumis sativus*) phloem sap (Hu *et al*., 2016), implying conservation of *TPS1* vascular expression across angiosperms. A conserved role for Tre6P within the vasculature would also align with the extensive expansion of *TPS*/*TPP* genes coinciding with the evolution of vascular tissues (Fichtner *et al*., 2021b).

Expression of class II *TPS* genes is less well understood. Single‐cell RNAseq studies show broad expression of many class II *TPSs* (Fig. 2), like *AtTPS8–10*, which are repressed by sucrose (Osuna *et al*., 2007). This group also includes *AtTPS9*, which is downregulated in growing axillary buds of pea and *Arabis alpina* (Vayssieres *et al*., 2020; Fichtner *et al*., 2024). However, some class II TPSs have more specialized expression patterns, like *AtTPS5*, which is confined to vascular tissues and the root cambium (Fig. 2) and is induced by sucrose but repressed by cytokinin (Osuna *et al*., 2007; Ramon *et al*., 2009). In rice, class II TPS proteins interact with OsTPS1 (Zang *et al*., 2011), while in arabidopsis, they associate with SnRK1 subunits, possibly modulating its localization (Van Leene *et al*., 2022). Moreover, several class II TPS are phosphorylated by SnRK1 and subsequently bind 14‐3‐3 proteins (Harthill *et al*., 2006). These findings suggest that class II TPS proteins may play a role in Tre6P signalling, either by modulating its synthesis rate or by binding to and signalling of Tre6P levels (Fig. 1b).

**Fig. 3:**
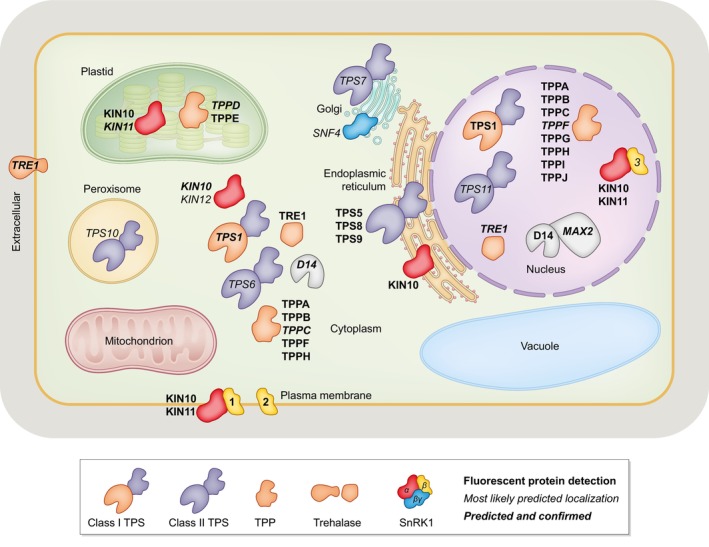
Intracellular localization of proteins involved in trehalose 6‐phosphate (Tre6P) biosynthesis and signalling in *Arabidopsis thaliana*. The localization of proteins marked in bold was shown by fluorescent protein tagging, while proteins in italics are based on the most likely localization predicted by SUBA5. Proteins highlighted in bold and italics have the same predicted and experimentally demonstrated localization pattern. D14, DWARF14; MAX2, MORE AXILLARY GROWTH2; SnRK1, Sucrose non‐Fermenting‐1‐Related Kinase 1; TPS, Tre6P synthases; TPP, Tre6P phosphatase; TRE1, TREHALASE1.

The *TPP* gene family in arabidopsis includes 10 members (*TPPA‐J*), all likely catalytically active (Vandesteene *et al*., 2012), and one gene encoding trehalase (*AtTRE1*). The *AtTRE1* gene encodes two isoforms: a membrane‐bound form with the catalytic domain oriented towards the apoplast, and a cytosolic/nuclear form (Fig. 3; Frison *et al*., 2007; Phan *et al*., 2022). Based on promoter GUS reporter fusion studies, *AtTPPs* and *AtTRE1* show distinct spatio‐temporal expression patterns: *AtTPPA* is broadly expressed, *AtTPPB/D/E/F/I/J* and *AtTRE1* are expressed in the shoot apex, *AtTPPD/H/I/J* in the root tip, *AtTPPH* and *AtTRE1* in the vasculature, and *AtTPPG* and *AtTRE1* in guard cells (Vandesteene *et al*., 2012). Single‐cell RNAseq studies confirmed the vascular expression of several *TPPs* (e.g. *TPPB/D/H/J*), and broader expression pattern of others (*TPPA/I*; Fig. 2). The broader expression of *TPPs* compared with *TPS1* suggests either Tre6P moves out of the vasculature to be dephosphorylated elsewhere, or that TPPs have additional functions. In maize, two TPPs modulate inflorescence branching independently of their catalytic activity (Claeys *et al*., 2019). A recent preprint reported the phenotype of a *10xAttpp* null mutant (Skopelitis *et al*., 2025), which showed only a mild increase in Tre6P levels and moderate transcriptomic changes, but increased branching and nutrient allocation defects, supporting potential roles for TPPs beyond Tre6P dephosphorylation.

Expression of *TPS1* overlaps with strigolactone signalling components, such as *MAX2* and the strigolactone receptor *DWARF14* (*D14*), expressed in the vasculature and meristems (Fig. 2). By contrast, SnRK1 subunits are more ubiquitously expressed, except for *KIN12* (*SnRK1α3*), which shows bundle sheath‐specific expression (Fig. 2b). Thus, for Tre6P to inhibit SnRK1 outside the vascular tissues, it would have to move cell to cell, for example, through plasmodesmata, which is likely given its small size (Tee & Faulkner, 2024).

## VIII. Intracellular localization of Tre6P signalling

Tre6P signalling likely takes place at multiple subcellular locations. Although experimental data are limited for most TPS and TPP proteins, AtTPS1 has been shown to localize to both the nucleus and the cytosol (Fichtner *et al*., 2020). Consistently, nonaqueous fractionation studies suggest that Tre6P levels are very low in chloroplasts and vacuoles (Martins *et al*., 2013). Using data from SUBA5 (Hooper *et al*., 2017, 2022) and fluorescent protein fusions (Chevalier *et al*., 2014; Krasensky *et al*., 2014; Williams *et al*., 2014; Blanco *et al*., 2019; Ramon *et al*., 2019; Struk *et al*., 2021; Van Leene *et al*., 2022), an overview of TPS, TPP, TRE1, SnRK1 subunits, D14, and MAX2 revealed a complex intracellular localization pattern (Fig. 3).

The class II TPS proteins AtTPS5/8/9 localize to the cytosol and endoplasmic reticulum (ER) when transiently expressed in *N. benthamiana* leaves (Van Leene *et al*., 2022), with the ER localization of TPS5 also confirmed by transient expression analysis using arabidopsis protoplasts (Tian *et al*., 2019). In young leaves, KIN10 itself is localized in the nucleus, but co‐expression with TPS5/8/9 redirects it to the ER (Van Leene *et al*., 2022). This dynamic movement of KIN10 between the nucleus and ER has also been observed in arabidopsis leaves (Blanco *et al*., 2019), and KIN10 has also been detected at the plasma membrane (Williams *et al*., 2014; Van Leene *et al*., 2022), and in chloroplasts (Fragoso *et al*., 2009; Ruiz‐Gayosso *et al*., 2018; Blanco *et al*., 2019). In arabidopsis root meristem cells, KIN10 is present in the nucleus but relocates to the cytoplasm in response to ABA (Belda‐Palazón *et al*., 2022).

The SnRK1 β1/2 subunits contain an N‐terminal myristoylation motif that anchors them at the plasma membrane, influencing KIN10 localization (Ramon *et al*., 2019). These findings suggest *in planta* Tre6P may inhibit KIN10 either directly in the nucleus or indirectly by altering its subcellular localization, which likely involved signalling via class II TPS proteins. For instance, class II TPS proteins may sequester KIN10 at the ER, reducing its nuclear or chloroplastic activity. This could explain Tre6P‐mediated inhibition of starch breakdown. However, transient expression in *N. benthamiana* leaves showed that two TPPs, AtTPPD/E, are localized in the chloroplasts (Krasensky *et al*., 2014) suggesting a direct function of Tre6P in the chloroplast. Krasensky *et al*. (2014) also showed that in *N. benthamiana*, AtTPPA/B/C/F/H localize to the cytosol and nucleus, while AtTPPG/I/J are exclusively nuclear‐localized (Fig. 3). The nuclear localization of several TPS/TPPs suggests that synthesis and degradation of Tre6P could occur directly in the nucleus, potentially enabling rapid transcriptional responses to fluctuations in Tre6P levels.

## IX. Conclusion

Tre6P has emerged as a central integrator of sugar availability with developmental decision‐making in plants. Acting both locally and systemically, Tre6P coordinates carbon allocation with growth through interactions with key regulators, such as SnRK1 and FT. Recent advances in single cell sequencing indicate a conserved vascular localization of TPS1. The intercellular and intracellular localization patterns of the Tre6P signalling nexus highlight the complexity of sugar signalling, both within a single organ and between different organs, as well as the subcellular localization of Tre6P pathway components. All of these layers of complexity must be considered when investigating Tre6P signalling. Tre6P is likely mobile between cells and may also move between tissues, as suggested by the broad expression of *TPPs* and the distribution of known Tre6P targets, such as SnRK1. Despite recent advances in our understanding of Tre6P signalling, key questions remain about the mechanisms of Tre6P mobility, its precise (sub)cellular sites of action, and its integration with hormonal and nutrient cues.

## Competing interests

None declared.

## Disclaimer

The New Phytologist Foundation remains neutral with regard to jurisdictional claims in maps and in any institutional affiliations.
